# Short-read and long-read full-length transcriptome of mouse neural stem cells across neurodevelopmental stages

**DOI:** 10.1038/s41597-022-01165-0

**Published:** 2022-03-02

**Authors:** Chaoqiong Ding, Xiang Yan, Mengying Xu, Ran Zhou, Yuancun Zhao, Dan Zhang, Zongyao Huang, Zhenzhong Pan, Peng Xiao, Huifang Li, Lu Chen, Yuan Wang

**Affiliations:** 1 Department of Neurosurgery, State Key Laboratory of Biotherapy and Cancer Center, West China Hospital, Sichuan University and National Collaborative Innovation Center, Chengdu, 610041 China; 2 Key Laboratory of Birth Defects and Related Diseases of Women and Children of MOE, State Key Laboratory of Biotherapy, West China Second Hospital, Sichuan University, Chengdu, 610041 China; 3 Core Facilities of West China Hospital, Sichuan University, Chengdu, China

**Keywords:** Neural stem cells, RNA sequencing

## Abstract

During brain development, neural stem cells (NSCs) undergo multiple fate-switches to generate various neuronal subtypes and glial cells, exhibiting distinct transcriptomic profiles at different stages. However, full-length transcriptomic datasets of NSCs across different neurodevelopmental stages under similar experimental settings are lacking, which is essential for uncovering stage-specific transcriptional and post-transcriptional mechanisms underlying the fate commitment of NSCs. Here, we report the full-length transcriptome of mouse NSCs at five different stages during embryonic and postnatal development. We used fluorescent-activated cell sorting (FACS) to isolate CD133^+^Blbp^+^ NSCs from C57BL/6 transgenic mice that express enhanced green fluorescent protein (EGFP) under the control of a Blbp promoter. By integrating short- and long-read full-length RNA-seq, we created a transcriptomic dataset of gene and isoform expression profiles in NSCs at embryonic days 15.5, 17.5, and postnatal days 1.5, 8, and 60. This dataset provides a detailed characterization of full-length transcripts in NSCs at distinct developmental stages, which could be used as a resource for the neuroscience community to study NSC fate determination, neural development, and disease.

## Abstract (summary)

**Table:** 

Measurement(s)	transcriptomic datasets of neural stem cells
Technology Type(s)	Next-Generation Sequencing(NGS) • third-generation full-length sequencing(TGS)
Sample Characteristic - Organism	Mus musculus
Sample Characteristic - Environment	laboratory facility
Sample Characteristic - Location	China

## Background & Summary

During mammalian brain development, neural stem cells (NSCs) give rise to major cell types in various brain regions, including neurons and glial cells (astrocytes and oligodendrocytes). Although embryonic and postnatal NSCs share molecular markers such as brain lipid-binding protein (Blbp, also known as fatty acid-binding protein 7, Fabp7) and CD133 (also known as Prominin-1), their cellular identities and fates vary significantly at different developmental stages. Embryonic NSCs are radial glial cells in the ventricular zone (VZ), which initially generate neurons in different cortical layers, and subsequently, undergo neuron-glia fate-switch to produce astrocytes and oligodendrocytes at late embryonic and perinatal stages^1^. After birth, a subset of radial glial cells transform into postnatal NSCs in the subventricular zone (SVZ) and subgranular zone (SGZ) in the hippocampus, which continue to generate interneurons and glia^2^. In the adult brain, the majority of NSCs in the SVZ and SGZ are committed to neuronal fate^3,4^. These fate switches in NSCs are driven by dramatic transcriptional alterations. Extensive efforts have been made to characterize human and mouse brain cells including NSCs at bulk and single-cell levels during neurodevelopment^5–7^. However, partly due to the scarcity of NSCs, full-length transcriptomic datasets of NSCs across different neurodevelopmental stages under similar experimental settings are lacking, which is essential for uncovering stage-specific transcriptional and post-transcriptional mechanisms underlying the fate commitment of NSCs.

Smart-seq2 is a powerful single-cell full-length sequencing protocol, which provides complete coverage across the genome allowing the detection of alternative transcript isoforms and SNPs^8,9^.

This protocol can also be adapted for full-length bulk RNA-seq of rare cell populations, such as NSCs. However, for the conventional 2^nd^-generation RNA-seq, cDNA generated from Smart-seq2 is fragmented before sequencing, resulting in accurate short-read raw data, which complicates the task of reconstructing and quantifying transcript isoforms. Long-read sequencing, or 3^rd^-generation sequencing, on the other hand, does not require cDNA fragmentation and provides a complete picture of the transcriptome at the cost of the sequencing accuracy. Combining short- and long-read sequencing can draw on their respective strengths.

**Fig. 1:**
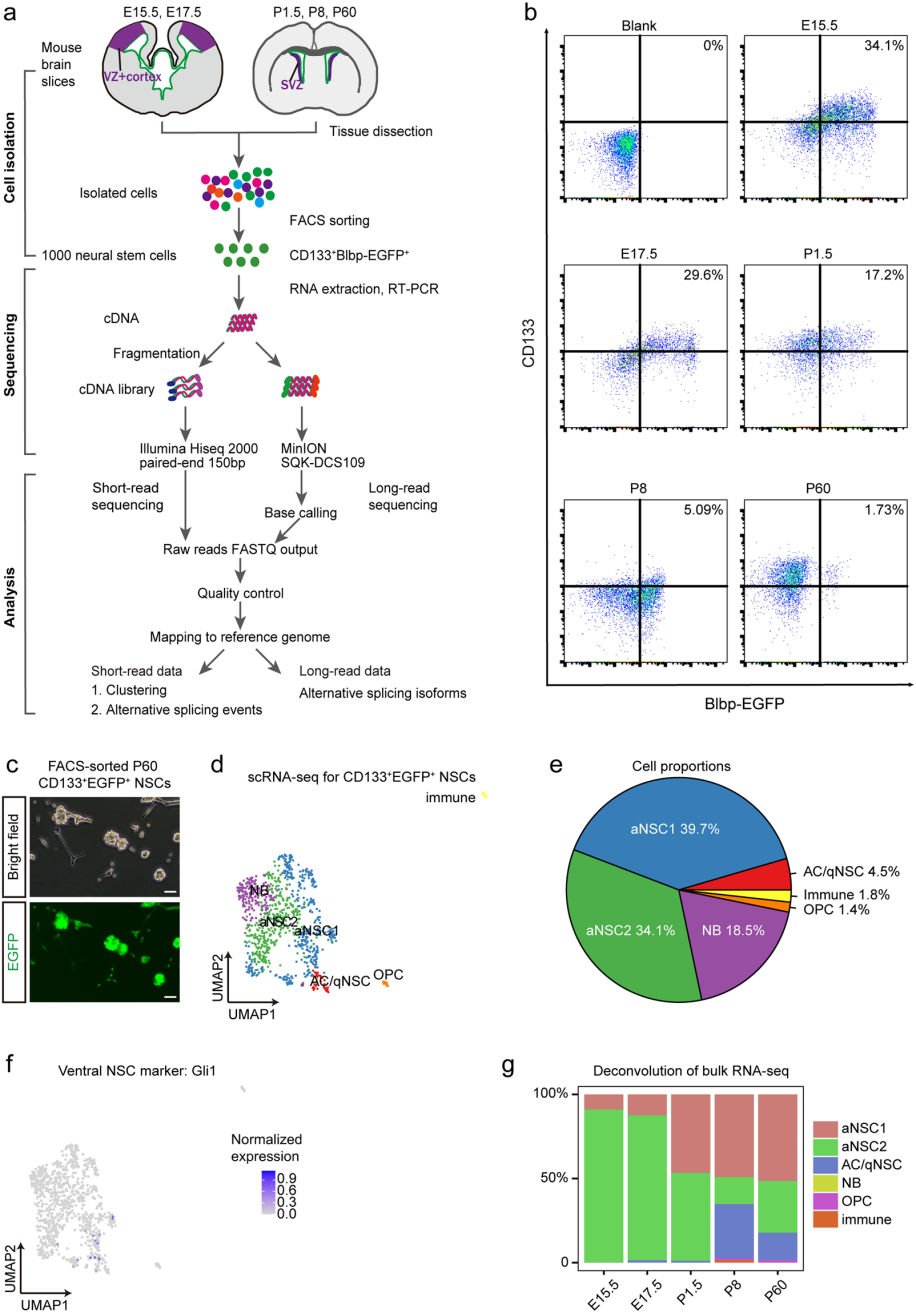
Study design, NSC isolation and validation. (**a**) The study design includes cell isolation, sequencing, and analysis. (**b**) Representative flow cytometric results of NSC sorting include blank samples and samples at each stage. (**c**) P60 CD133^+^Blbp-EGFP^+^ cells from FACS were cultured into neurosphere after 4-day *in vitro* and carried green fluorescence EGFP. Scale bar, 100 μm. (**d**) UMAP analysis integrating high-quality single cells from sorted NSCs samples at P60. Clusters are separated by colours. (**e**) The Pie chart shows the proportion of each cell type by UMAP clusters. (**f**) UMAP shows the expression of Gli1, a marker for ventral NSCs, in the NSC populations. (**g**) Deconvolution of bulk RNA-seq. The percentage of cell types at five time points.

In this study, we used fluorescence-activated cell sorting (FACS) to isolate CD133^+^Blbp-EGFP^+^ NSCs from C57BL/6 transgenic mice at five different stages of embryonic and postnatal development, including embryonic day 15.5 (E15.5, the peak of cortical neurogenesis), E17.5 (the transition to gliogenesis), postnatal day 1.5 (P1.5, neonatal stage), P8 (the peak of postnatal NSC proliferation and gliogenesis), and P60 (adult). We used the Smart-seq2 protocol to prepare the cDNA samples of NSCs at these stages, and performed a total of 20 short-read RNA-seq with at least three samples per stage, along with paired Oxford Nanopore long-read RNA-seq for each stage. The whole study design of the present study is present in Fig. 1a. The resultant dataset provides a detailed characterization of full-length transcripts in NSCs at distinct developmental stages in a similar experimental setting and could be used as a resource to study NSC fate determination, neural development, and disease.

## Methods

### Animals

The Blbp-EGFP mice used in this study were initially generated by Anthony *et al*. at The Rockefeller University and obtained from Dr. Yuan Zhu’s lab at Children’s National Medical Center in Washington, DC under a material transfer agreement with Sichuan University. The mice were bred in the Experimental Animal Centre of Sichuan University and maintained on a C57BL/6 genetic background. Mice were housed in pressurized, individually ventilated cages (PIV/IVC) and maintained under specific-pathogen-free conditions, with free access to food and water in a 12 h light/dark cycle. All animal studies were approved by the Animal Care and Use Committee of Sichuan University. For timed pregnancies, the plug date was designated as E0.5 and the date of birth was defined as P0.5.

### Sample collection and FACS

To collect embryonic NSCs, embryonic brains were placed into pre-chilled 10% FBS solution (10% FBS in DPBS, Gibco), and the dorsal wall of the LV was dissected out under a dissecting microscope (Motic). The tissue was dissociated by pipetting, and the cells were filtered through a 40 µm nylon mesh cell strainer (BD Falcon) to prepare single-cell suspension.

Postnatal brains were placed into 10% FBS solution, cut into coronal slices, and the SVZ region was harvested, minced into small pieces, and dissociated with Accutase solution (Millipore) at 37 °C for 20 min. The resultant cells were filtered through a 40 µm nylon mesh cell strainer (BD Falcon) to prepare single-cell suspension.

NSCs were stained with viability marker fixable viability stain 510 (FVS510, BD Horizon, 564406) and NSC marking antibody CD133-APC (Abcam, ab19898), and subjected to FACS. 1000 FVS510^−^/CD133^+^/Blbp-EGFP^+^ cells from each mouse were collected for subsequent RNA-seq (Fig. 1b). The FACS plots for all samples are presented in Figshare^10^. The cells for 10X single-cell RNA sequencing are also derived from FACS with the same sorting strategy as bulk RNA-seq.

### NSC Culture

Sorted FVS510^−^/CD133^+^/Blbp-EGFP^+^ cells were cultured in NSC culture medium (1% N2, 2% serum-free B27, 20 ng/ml EGF and 20 ng/ml bFGF in DMEM/F12) in 6-well ultra-low binding plates (Corning). Neurospheres are visible after 4 days of culture. (Fig. 1c).

### cDNA library construction and sequencing

#### cDNA preparation

cDNA preparation was modified from a published protocol which was originally used for single-cell RNA sequencing^11^. Briefly, all the components of lysis buffer (TritonX-100, dNTP, Oligo-dT VN primer, and RNase inhibitor) were 2X except RNase inhibitor which was increased to 8X,resulting in a total volume of 8.8 µL. The components for RT-PCR reaction mix were increased accordingly. NSCs were collected in tubes containing lysis buffer and were immediately transferred onto dry ice. The lysate was vortexed vigorously for 1 min followed by incubation at 72°C for 3 min, and subjected to RT-PCR. Reverse transcription mixture was prepared by mixing 1.6 µL SuperScript II reverse transcriptase, 1.6 µL RNase inhibitor, 6.5 µL Superscript II first-strand buffer, 1.6 µL DTT, 6.5 µL betaine, 0.2 µL MgCl_2_, 0.3 µL TSO and 0.9 µL nuclease-free H_2_O to reach a total volume of 19.2 µL. Cell lysate was mixed with reverse transcription mixture and incubated at 42°C for 90 min, followed by 10X RT-PCR cycles: ① 50 °C for 2 minutes ②42 °C for 2 minutes. Afterwards, the reverse transcribed cDNA samples were incubated at 70 °C for 15 min. For additional PCR amplification, 33.5 µL cDNA was mixed with 33.5 µL KAPA HiFi HotStart ReadyMix and 0.7 µL ISPCR primers to a total volume of 67.7 µL. The mixture was first incubated at ③ 98 °C for 3 minutes, followed by 20X PCR cycles: ① 98 °C for 20 seconds, ② 67 °C for 15 seconds and ③ 72 °C for 6 minutes. The amplified cDNA samples were incubated at 72 °C for 5 minutes. cDNA purification was carried out with Ampure XP magnetic beads (0.8:1 ratio, Beckman Coulter, A63881). Before library construction, cDNA quality was checked with Agilent 2100 Bioanalyzer (Invitrogen). Library construction was performed with qualified cDNA for both short-read and long-read sequencing. The 10X single cell RNA-seq were prepared in the Chromium Single Cell Gene Expression Solution using the Chromium Single Cell 3′ Gel Bead, Chip and Library Kits v2 (10X Genomics) as per the manufacturer’s protocol. 8000–10,000 total cells were added to each channel. The cells were then partitioned into Gel Beads in Emulsion in the Chromium instrument, where cell lysis and barcoded reverse transcription of RNA occurred, followed by amplification, shearing 5′ adapter, and sample index attachment. Libraries were sequenced on the Illumina NovaSeq 6000 platform at Novogene, Beijing, China^12^.

#### Short-read sequencing

Qualified cDNA samples were respectively taken for short-read library construction, including DNA fragmentation, end-repair, 3′ ends A-tailing, adapter ligation, PCR amplification, and library validation. cDNA library was subjected to quality inspection with PerkinElmer LabChip® GX Touch. Qualified libraries were then loaded on the Illumina Hiseq platform for PE150 sequencing.

#### Long-read sequencing

The cDNA samples for long-read sequencing were taken from the same pools for short-read sequencing. Equal content of cDNA samples of the same stage were mixed into one sample. ONT Ligation Sequencing Kit (SQK-LSK109) was used for library preparation according to the manufacturer’s instructions except that DNA was not sheared before native barcode ligation. The library construction included DNA repair, end preparation, native barcode ligation, purification pooling, and sequencing adapter ligation. The cDNA libraries were pooled evenly at the amount of 80 ng from each stage. Sequencing was performed using MinKNOW (v20.06.4, Oxford Nanopore Technologies Ltd.). MinKNOW is the instrument control software that runs on the host computer to which the MinION equipped with an R9.4.1 flow cell is connected. The data output from MinKNOW consist of 4,000 sequence reads in an HDF5 format called FAST5.

#### Validation of splice junctions by PCR

To validate NSCs time points-specific splice junctions we designed exon-specific PCR primers for 8 stage-specific alternative splicing events, including two types of splice junctions: Alternative 3′splice site (A3SS) and Skipping exon (SE). Pooled cDNA libraries for NSCs from each time point (E15.5, E17.5, P1.5, P8 and P60) were mixed with the primers and subjected to 30X PCR cycles. Each PCR reaction contained 25 ng of library DNA template and 10 pmol of each gene specific primer in a PCR master mix (2X Phusion Plus Green, Thermo Scientific) at a total volume of 50 µl. PCR products were subjected to electrophoresis separation in 2% TBE/agarose gels. The images were captured and gel band intensity was calculated by Image J. The PCR PSI is calculated as the intensity of the long transcript divided by the total intensity of the long + short transcripts.

**Table:** 

Gene Name	Forward Primer (5′→3′)	Reverse Primer (5′→3′)
***Capzb***	GCACGCTGAATGAGATCTAC	GCGTGGTCGATGCAAACTG
***Dync1i2***	CTATGTCTCCATCCTCCAAGTC	GGTCTGAGTTTCCTTTGTGTATG
***Gkap1***	CTCCCGCTCCAGAGCACAAC	GGACCGTCAGCTCGTTCTTG
***Gpm6b***	CATGTCCTATCACCTGTTCATTG	CAGTTCCTGCTCTTCCTTTGC
***Hnrnpdl***	CCAGAACAATTACCAGCCCTAC	GAGTCATCATAACACAGGTAGC
***Nkain4***	GTCTATGGTTGCTACGTGGTCAG	CTCACAGTTGTAGCCACCCTGTC
***Abat***	GAGAACGGTGGCTGGAATCATCG	GCAGGTCTTCCCGCTTGATGATG
***Sox5***	CACCAGGCTTAGGCCCACTC	CAGAGCTGGCATGTGAGGAGAG

### Data processing

#### Deconvolution of bulk RNA-seq

We used MuSiC to deconvolute the transcriptome of Bulk RNA-Seq samples into the likely constituent cell types, using scRNA-seq datasets from same samples as Bulk RNA-Seq as a reference. We calculated the predicted proportions of each cell type in bulk samples, and visualized these proportions with bar plot^12^.

#### ScRNA-seq data analysis

We used Seurat (v3.1.0) for downstream analyses including data normalization (NormalizeData, LogNormalize method, scaling factor 10,000), data feature scaling (ScaleData), variable gene detection (FindVariableGenes with vst method) and PCA of variable genes (RunPCA). The statistically significant PCs were used for Harmony to remove the batch effect, and the two-dimension UMAP was calculated among the Harmony matrix^13^. Then the original Louvain algorithm (FindClusters) with clustering resolution 1.4 was performed to cluster the cells. We computed DEGs using the FindAllMarkers function in the Seurat package with default parameters. To determine the cell types, we used the list of DEGs and the published dataset of marker genes^10,14^.

#### Base calling

The raw data generated by MinKNOW software were converted from.fast5 files to base-called.fastq files under high accuracy mode using the ONT basecaller Guppy software (v.4.0.14)^15^. In the meantime, the sample barcodes were trimmed off with the modes ‘--barcode_kits’ and ‘--trim_barcode’.

#### Quality control

The quality of the short-read sequencing data was checked using FastQC software (v0.11.8) (http://www.bioinformatics.babraham.ac.uk/projects/fastqc/) and RSeQC package (v4.0.0)^16^ (http://rseqc.sourceforge.net). For long-read sequencing data, the quality check was performed with NanoComp software (v1.33.1)^17^.

#### Alignment

The paired-end reads of short-read sequencing were aligned to the mouse reference genome GRCm38 with annotation from ENSEMBLE release 93 using STAR (v2.7.1a)^18^, and the reads of long-read sequencing were aligned to the same genome file using Minimap2 (v2.17-r974-dirty)^19^.

#### Aligned reads distribution

Gene body coverage, reads’ distribution over genome feature, and RNA integrity at cDNA level of short-read sequencing data were calculated by geneBody_coverage.py, read_distribution.py, and tin.py from RSeQC^16^, respectively.

#### Gene expression quantification

For short-read sequencing data, the gene expression was quantified using the HTSeq (v0.11.2)^20^. The raw read counts were then normalized by their library size factors and were normalized to stabilize the variance across the samples using DESeq 2 (v1.28.1)^21^ with variance stabilizing transformation (VST). The top 500 highly variable genes were utilized for unsupervised clustering analysis.

#### Differential splicing usage

The percent-spliced-in (PSI, also denoted Ψ) value was calculated according to a previous study^22^ and recapitulated here. The PSI metric was computed directly by counting reads that aligned to known or predicted splicing junctions (SJs) generated from STAR^18^. The significance of enrichment was tested by a two-tailed hypergeometric test^23^. The functional annotations of each AS event were performed by using the GenomicRanges R package (v1.40.0, findOverlaps module)^24^ and mouse reference genome GRCm38 with annotation from ENSEMBLE release 93.

#### Identification of AS modes

The resulting alignments (in BAM format) of long-read sequencing data were used to build sample-specific transcriptome assembled with Stringtie2 (v2.1.4)^25^. The mode -G was specified to use the mouse reference, and the mode -L was specified in long reads format. GffCompare^26^ was used to compare and evaluate the accuracy of Stingtie2^25^ transcript assemblers. The consolidated set of accurate isoforms (GTF format) were used to obtain a list of all possible AS events, and SUPPA2 generateEvents mode^23^ was used to generate all AS events with the parameter of “-f ioe -e SE SS MX RI FL”.

#### Transcript visualization

For visualization of transcripts, samtools (v1.10.2)^27^ was used to extract the gene region, bedtools (v2.30.0)^28^ was used to convert bam file to bed format files, UCSC tools were used to convert bed format files to GTF format. Visualization was carried out by using R package ggbio (v1.36.0)^29^. Sashimi plots of short-read sequencing data were plotted using pysashimi (https://github.com/ygidtu/pysashimi).

## Data Records

The raw fastq files were deposited at NCBI under accession number SRP321063^30^. The FACS data for individual samples, as well as the processed files, including the quantification of gene expression, isoforms, SJs from both short-read and long-read data were uploaded in Figshare^10^. We also included detailed gene expression matrix and cell type determination of a validation scRNA-seq dataset in Figshare^10^.

## Technical Validation

### NSC purity

To confirm our sorted cells are indeed mainly composed of NSCs, we first performed neurosphere culture assay. Sorted cells readily formed Blbp-EGFP^+^ neurospheres after 4-day non-adherent culture *in vitro*, indicating they are neural stem or progenitor cells (Fig. 1c). We further performed single-cell RNA-seq on CD133^+^Blbp-EGFP^+^ cells from P60 SVZ to identify individual cell types in these cells^10^. The majority of the cells are aNSCs (73.8%) (Fig. 1d,e). The qNSC population is relatively minor, and is transcriptionally hard to distinguish from astrocytes. Consistent with our sampling of dorsal SVZ regions, Gli1, a marker for ventral NSCs, is barely detected in the NSC populations (Fig. 1f). To determine the NSC percentage in bulk samples, we performed deconvolution of bulk RNA-seq using our scRNA-seq dataset as a reference. Consistently, the majority of the cells are aNSC-like (Fig. 1g). These data support that our sample collection method can enrich for NSCs.

### RNA integrity

As the mRNA was reversely transcribed immediately after bulk NSCs were lysed, the mRNA integrity could not be measured directly. We performed the quality check by examining the fragment distribution of cDNA. It turned out that all peaks of the sample cDNA were longer than 1200 bp (Online-only Table 1). The RNA integrity at the transcript level was further evaluated using the Transcript Integrity Number (TIN) algorithm, which was calculated with the tin.py script from the RSeQC package^16^. TIN represents a score ranging from 0 to 100 for each expressed transcript, and the medTIN (median TIN score across all the transcripts) can be used to measure the RNA integrity at the sample level. The mean TIN score of all samples was 43.87 (Online-only Table 1).

### Data quality

**Fig. 2:**
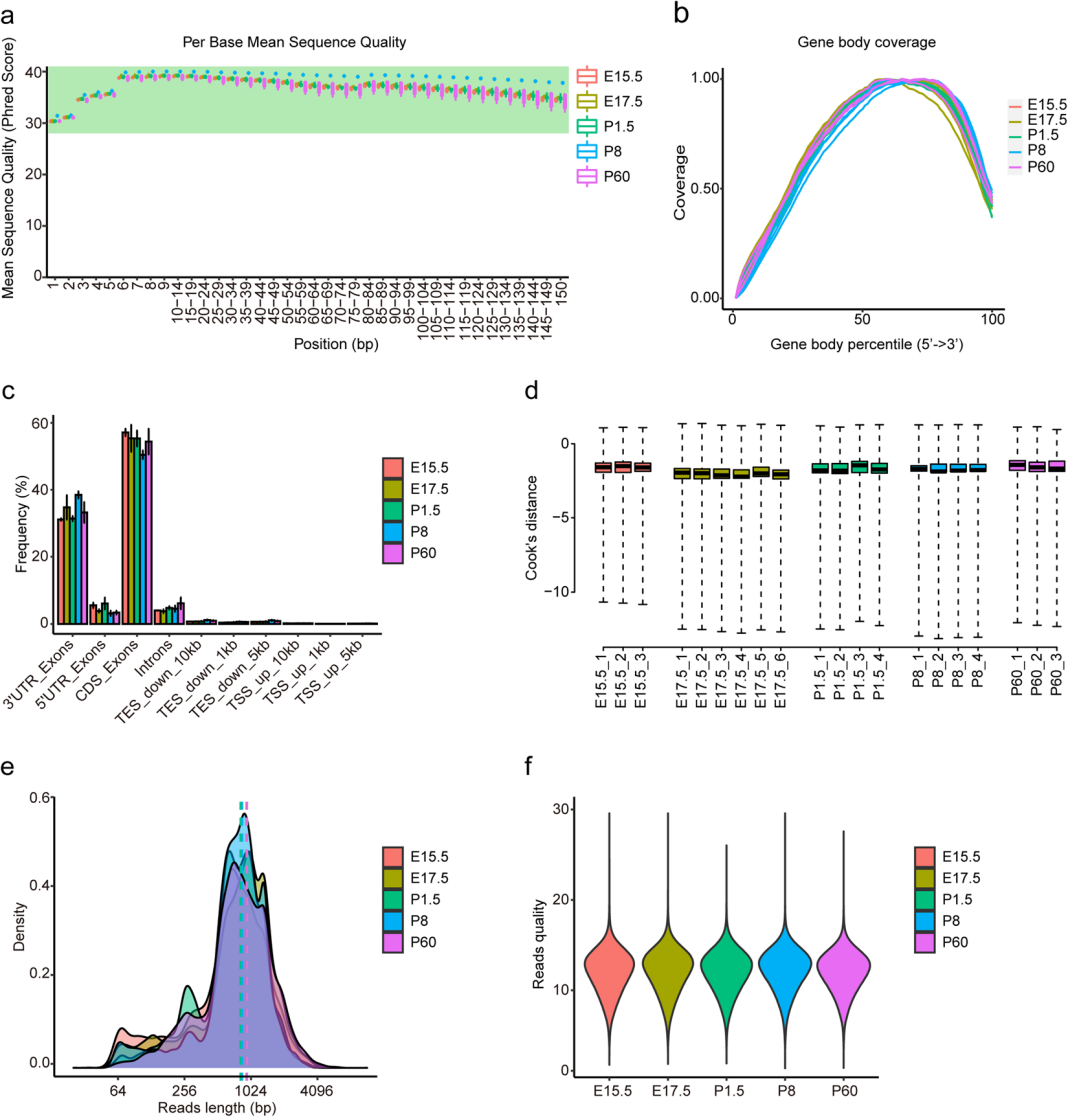
Quality control of short-read and long-read sequencing data. (**a**) Average sequencing quality per base of each sample. (**b**) Reads distribution along the gene body. (**c**) The frequency of counts in various gene regions and the error bars depict the standard deviation. (**d**) Cook’s distance was calculated for each sample. (**e**) Distribution of reads length of randomly selected 50,000 reads from all samples. (**f**) Base calling quality score of each sample.

Biological replicates are fundamental to guarantee data reliability. In the present study, we took 3 E15.5 samples, 6 E17.5 samples, 4 P1.5 samples, 4 P8 samples, and 3 P60 samples for bulk transcriptome sequencing. The average depth was 25.99 M (SD = 11.78) for the short-read sequencing. The quality of each base generated was assessed using FastQC. There is no significant difference in the distribution of average quality score per base in samples from different stages, and the mean of Q30 is over 100% (Fig. 2a). The reads generated from all samples were distributed approximately uniform across the gene body (Fig. 2b). We further gathered the gene regions where the reads mapped to, and more than 90% of reads were mapped to exon regions (Fig. 2c). Moreover, Cook’s distance was calculated to test for outliers, with none detected (Fig. 2d). All the samples have over 80% uniquely mapped reads (Online-only Table 1). Besides, Q30 of each sample is higher than 85% (Online-only Table 1).

For long-read sequencing data, we selected 20.6 Gb reads using Guppy^15^ from 77 Gb raw data and identified 12,387,984 reads. The sequencing statistics were counted using NanoComp^17^. The mean read length of all the five stages was around 800 bp, and the P60 sample had the maximum mean length (Fig. 2e, Online-only Table 1). On the aspect of base calling quality, the mean read quality score was above 12 for each sample (Fig. 2f, Online-only Table 1).

**Fig. 3:**
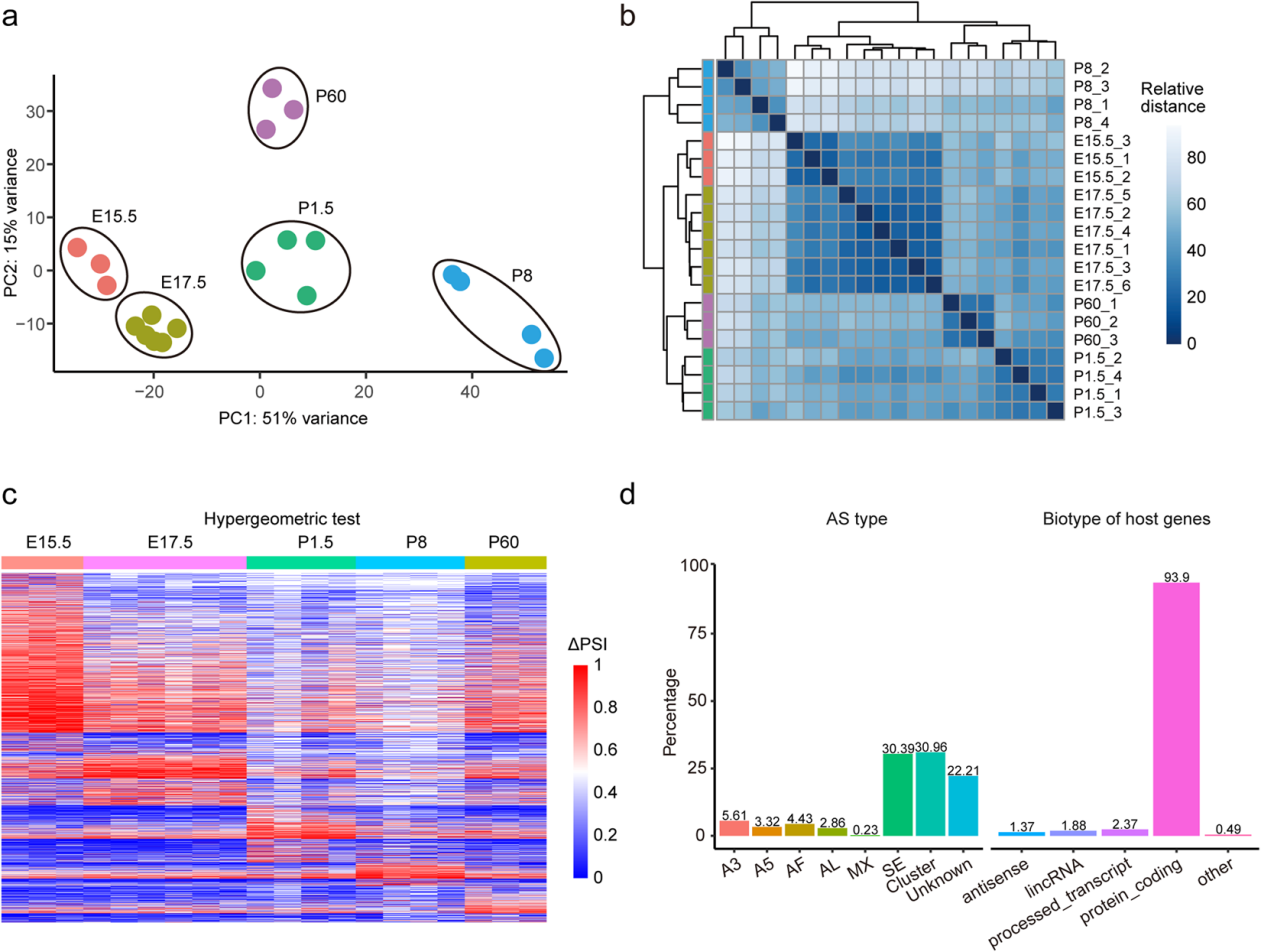
Samples clustered by gene expression and the differential splicing usage based on short-read sequencing. (**a**) PCA analysis for all samples. (**b**) Hierarchical clustering analyses for each sample. (**c**) Heat-map visualizing the differential SJs’ PSIs in samples from each stage. (**d**) The proportion of AS types and biotypes of the host genes for differential SJs.

To establish the congruency of short-read data among all stages, we carried out principal component analysis (PCA) (Fig. 3a) and hierarchical clustering (Fig. 3b) using the top 500 highly variable genes from normalized RNA-seq data with variance stabilizing transformation (VST) in DESeq 2^21^. The PC1 explained 51% of the variance, while the PC2 explained 15% variance. PCA revealed that stage E15.5 was close to E17.5, while stage P1.5 was close to P60 (Fig. 3a). Besides, the samples of P8 were distant from all the other stages (Fig. 3a). Hierarchical clustering showed a similar result to that of PCA (Fig. 3b).

### Differential splicing usage

As an index of AS, the PSI value was calculated for the inclusion levels of internal exons, as described in a previous study^22^. Differential PSI was calculated via a two-tailed hypergeometric test. The heatmap (Fig. 3c) showed all the 4403 differential alternative SJs in 5 stages with the P-value < 0.01, ΔPSI > 0.2, and these SJs were detected in more than 60% samples of a target group and existed in other groups in short-read sequencing data. To further understand the differential splicing usage, we analyzed the AS types and the host genes of differential SJs. The SJs with single skipping exon (SE) (30.39%) were much more than SJs with other singles, including alternative 3′ splice site (A3), alternative 5′ splice site (A5), alternative first exon (AF), alternative last exon (AL) and mutually exclusive exons (MX) (Fig. 3d). Besides, there were 22.21% SJs with unknown AS types and clusters (multi-AS types). Most host genes (93.9%) were protein-coding genes, with only 2.37% host genes expressing processed transcripts, 1.88% expressing lincRNA, and 1.37% as antisense genes (Fig. 3d). The left 0.49% host genes belong to other types (Fig. 3d).

### Stage-specific AS

**Fig. 4:**
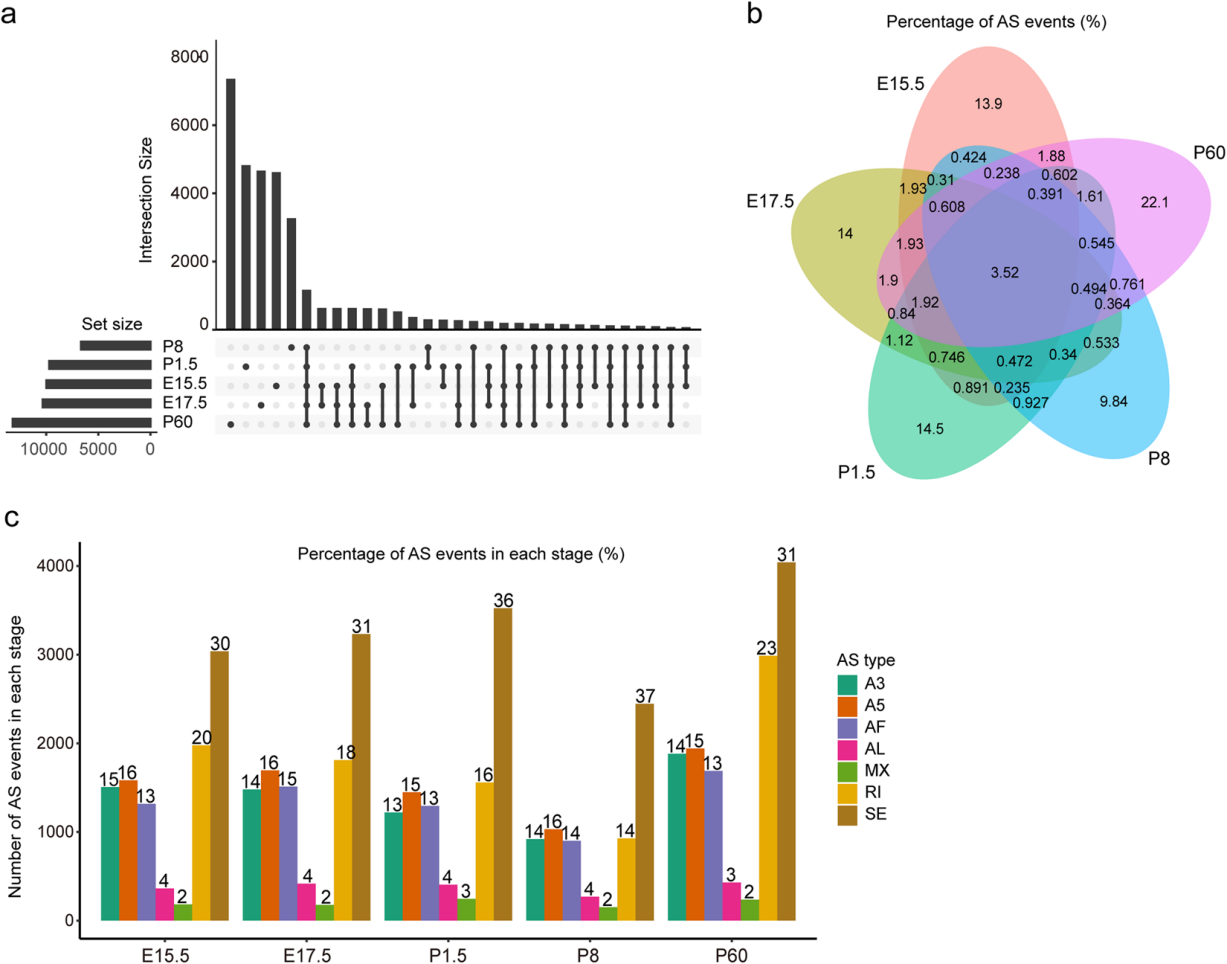
AS event analysis based on long-read sequencing data. (**a**) The upset plot shows the numbers of specific and total AS events in all stages. (**b**) Venn diagram shows the percentages of specific and overlapped AS events in all stages. (**c**) The numbers (y-axes) and percentages (above the histogram) of detected AS types in each stage.

For long-read sequencing data, seven types of AS were quantified to analyse the relative contribution of AS at all five stages. The quantification of each type of AS event was performed by using the SUPPA2 program^31^. In total, 33,230 AS events were identified from all five stages. The P60 stage had the most 13211 AS events, while P8 had the least 6649 AS events (Fig. 4a). Moreover, both E15.5 and E17.5 had nearly 10,000 AS events (Fig. 4a). Stage E15.5 and E17.5 had the most overlapped AS events (Fig. 4a). The P60 stage had the largest percentage of specific AS events (22.1%), while P8 had the smallest percentage of specific AS events (9.84%) (Fig. 4b). The other 3 stages had around 14% specific AS events (Fig. 4b). The percentage of each AS type was also analyzed. SE was the most AS type, which constitutes 30%~37% AS events in each stage, whereas AL and MX were the least with each frequency less than 5% (Fig. 4c).

**Fig. 5:**
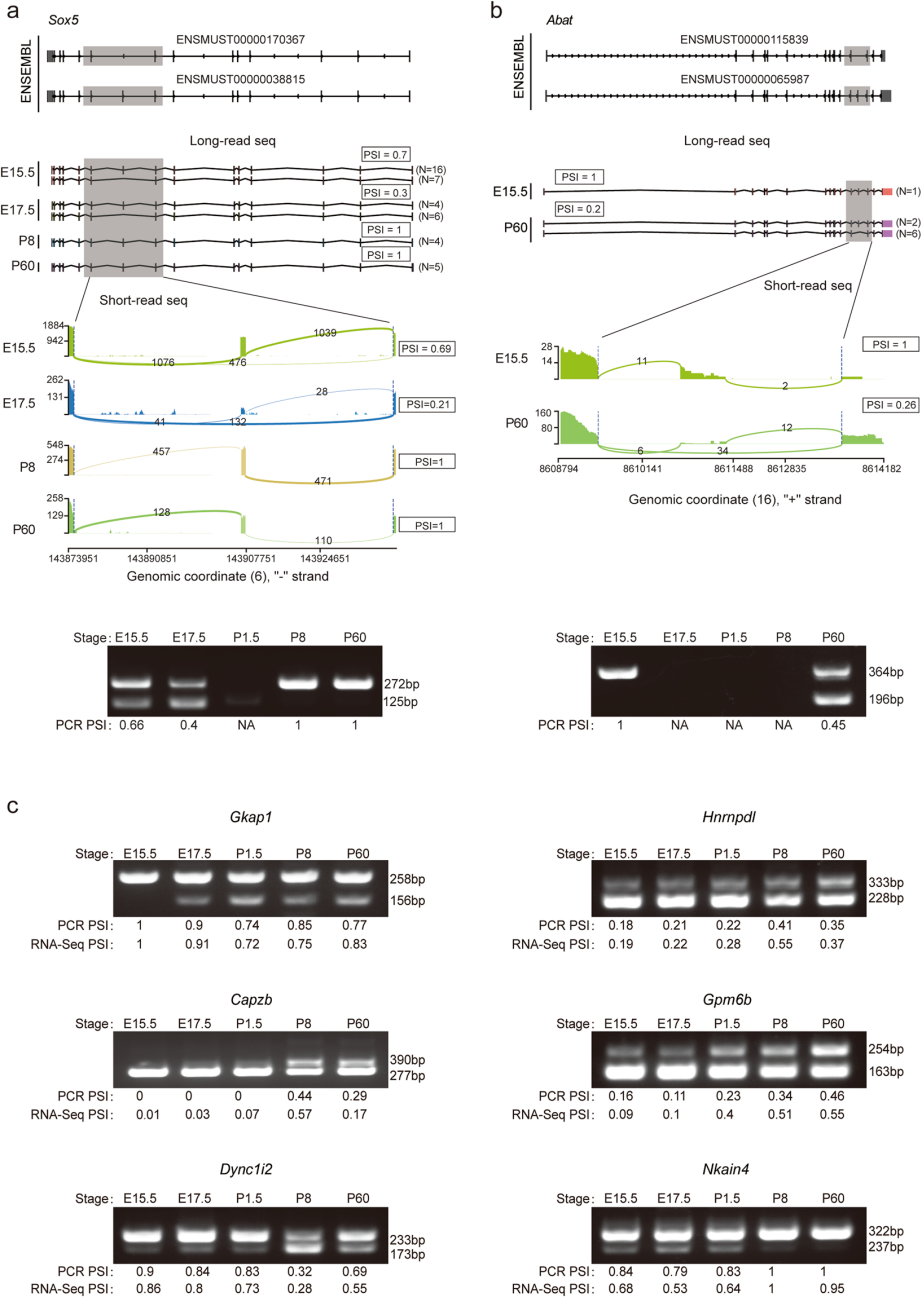
Transcript visualization and PCR validation of AS. (**a,b**) Transcript visualization of gene *Sox5* and *Abat*. Upper panels, transcript annotations in ENSEMBL. Middle panels, reads from long-read and short-read sequencing by stage. PSI represents the percentage of splicing, and N represents the counts of reads. Lower panels, PCR validation of AS events. The PCR PSIs were calculated and shown. (**c**) PCR validation for 6 additional alternative splicing events (*Gkap1, Hnrnpdl, Capzb, Gpm6b, Dync1i2 and Nkain4*), and the PCR PSIs are consistently correlated with RNA-seq PSIs.

To further test the consistency between short-read and long-read sequencing data, we checked the SE of two neural development-associated genes, *Sox5* and *Abat* (Fig. 5a). We screened some reads according to their start and end sites within the range of 600 bp upstream and downstream of the annotated transcripts in the mouse reference genome GRCm38. For *Sox5*, Exon 7 skipping was annotated by Ensembl which involves 2 transcripts, ENSMUST00000170367 and ENSMUST00000038815 (Fig. 5a). These 2 transcripts were found out in the long-read sequencing data but were not evenly distributed among the five stages. These transcripts were highest in E15.5 samples and not expressed in P1.5 samples (Fig. 5a). Samples from embryonic stages (E15.5 and E17.5) but not postnatal stages contain the transcript that skips Exon 7 (Fig. 5a). The splicing junction analysis of short-read sequencing data showed that Exon 7 skipping occurred partially at E15.5 (PSI = 0.69) and E17.5 (PSI = 0.21) stages (Fig. 5a). However, no Exon 7 skipping occurred at P8 (PSI = 1) or P60 stages (PSI = 1) (Fig. 5a). For gene *Abat*, Exon 12 skipping was annotated by Ensembl which involves 2 transcripts, ENSMUST00000115839 and ENSMUST00000065987 (Fig. 5b). The former transcript with no Exon 12 was only found in long-read sequencing data of the P60 stage, while the later one harbouring Exon 12 was found in both E15.5 and P60 stages (Fig. 5b). Splicing junction analysis of short-read sequencing also found that Exon 12 skipping occurred at the P60 stage (PSI = 0.26) but not at E15.5 (PSI = 1) The exon retention ratios for *Sox5, Abat* are confirmed by PCR analysis (Fig. 5a,b). We performed PCR validation for 6 additional alternative splicing events (*Gkap1, Hnrnpdl, Capzb, Gpm6b, Dync1i2* and *Nkain4*), and the PCR PSIs are consistently correlated with RNA-seq PSIs (Fig. 5c). Detailed transcript information for these genes are available in Figshare^10^.

## Usage Note

The present study provides full-length transcriptomic profiles of mouse NSCs across embryonic and postnatal stages. As the dataset contains both short-read and long-read sequencing data, the profiles are reliable for related researches. The profiles are valuable for transcriptional and posttranscriptional mechanisms of neurodevelopment and fate commitment of NSCs, especially the stage-specific gene expression and alternative splicing. Besides, the profiles are also suitable for exploring the molecular mechanisms underlying diseases related to neurodevelopment.

## Data Availability

The codes used in this article were deposited in https://github.com/LuChenLab/Neuron.

## Online-only Table

**Online-only Table 1:** Sample and sequencing information.

Stage	Sample ID	cDNA peak (bp)	Short-read data						Long-read data		
			Clean reads (Million)	GC (%)	Q30 (%)	TIN (median)	Uniquely mapped reads (%)	Average mapped length (bp)	Mean read length (bp)	Number of reads (Million)	>Q7
E15.5	E15.5_1	1282	19.3	43	90.7	50.6	86.8	295.37	754	2.3	98.10%
	E15.5_2	1310	21.6	44	90.7	51.8	88.46	294.78			
	E15.5_3	1399	18	45	90.5	51.8	89.67	295.15			
E17.5	E17.5_1	1393	28.5	42	91.2	45.4	86.41	295.46	845.8	2.8	98.00%
	E17.5_2	1384	26.3	42	90.9	45.9	86.08	295.46			
	E17.5_3	1378	17.7	42.5	88.4	50.8	83.47	293.53			
	E17.5_4	1388	15.7	42	90.8	45.5	83.81	295.53			
	E17.5_5	1342	25	44	90.8	49	86.57	295.08			
	E17.5_6	1316	22.9	42.5	92.9	49.4	84.36	295.56			
P1.5	P1.5_1	1299	14.5	41	91.3	36	82.34	294.51	747	2.2	98.50%
	P1.5_2	1358	13.9	41	91.2	34.9	82.76	294.51			
	P1.5_3	1402	33.2	42	92.4	41.7	86.2	295.54			
	P1.5_4	1334	19.2	43	89.6	48.2	82.43	293.55			
P8	P8_1	1311	44.7	42	94.8	32.2	90.02	298.13	782.6	2.7	98.40%
	P8_2	1309	45.6	40	95.4	25	86.61	298.3			
	P8_3	1294	45.1	40	95.5	26.5	86.04	298.35			
	P8_4	1307	44.3	42	95	33.2	91.77	298.29			
P60	P60_1	1833	31.5	44	90.8	51.1	89.7	295.36	856.7	2.4	98.50%
	P60_2	1817	26.3	44	91.4	53.1	89.59	296.78			
	P60_3	1394	14.6	43	86.6	55.3	84.71	291.35			
